# Acute Myeloid Leukemia Genome Characterization Study and Subtype Classification Employing Feature Selection and Bayesian Networks

**DOI:** 10.3390/biomedicines13051067

**Published:** 2025-04-28

**Authors:** Zhenzhen Li, Jingwen Li, Sifan Li, Yangyang Wang, Jihan Wang

**Affiliations:** 1 Research & Development Institute of Northwestern Polytechnical University in Shenzhen, Shenzhen 518057, China; lizhenzhen@nwpu.edu.cn (Z.L.); lijingwen@mail.nwpu.edu.cn (J.L.); lisifan@mail.nwpu.edu.cn (S.L.); 2 Xi’an Key Laboratory of Stem Cell and Regenerative Medicine, Institute of Medical Research, Northwestern Polytechnical University, Xi’an 710072, China; 3 School of Physics and Electronic Information, Yan’an University, Yan’an 716000, China; 4 Yan’an Medical College, Yan’an University, Yan’an 716000, China

**Keywords:** AML, feature selection, classification, subtypes

## Abstract

**Background:** The precise diagnosis and classification of acute myeloid leukemia (AML) has important implications for clinical management and medical research. **Methods:** We investigated the expression of protein-coding genes in blood samples from AML patients and controls using The Cancer Genome Atlas (TCGA) and Genotype-Tissue Expression (GTEx) databases. Subsequently, we applied the feature selection method of the least absolute shrinkage and selection operator (LASSO) to select the optimal gene subset for classifying AML patients and controls as well as between a particular FAB subtype and other subtypes of AML. **Results:** Using LASSO method, we identified a subset of 101 genes that could effectively distinguish between AML patients and control individuals; these genes included 70 up-regulated and 31 down-regulated genes in AML. Functional annotation and pathway analysis indicated the involvement of these genes in RNA-related pathways, which was also consistent with the epigenetic changes observed in AML. Results from survival analysis revealed that several genes are correlated with the overall survival in AML patients. Additionally, LASSO-based gene subset analysis successfully revealed differences between certain AML subtypes, providing valuable insights into subtype-specific molecular mechanisms and differentiation therapy. **Conclusions:** This study demonstrated the application of machine learning in genomic data analysis for identifying gene subsets relevant to AML diagnosis and classification, which could aid in improving the understanding of the molecular landscape of AML. The identification of survival-related genes and subtype-specific markers may lead to the identification of novel targets for personalized medicine in the treatment of AML.

## 1. Introduction

AML is the most prevalent leukemia among the adult population and accounts for approximately 80% of all cases. It is an extremely aggressive and heterogeneous disorder characterized by the expansion of myeloid precursors in the bone marrow that are arrested in the early stages of development. It is thought to originate from the defective regulation of the differentiation and self-renewal programs of primitive multipotent hematopoietic stem cells (HSCs) or progenitor cells as a result of chromosomal translocations, genetic mutations, and changes at the molecular level [1,2]. According to the new 5th World Health Organization (WHO) classification of hematolymphoid tumors, AML is classified into two families according to morphology, cytogenetics, molecular genetics, and immunological markers: AML with defining genetic abnormalities and AML defined by differentiation [3]. Although genetic abnormalities are one of the most useful classification indicators in AML, about 50% of de novo AML patients have normal karyotypes; these patients lack the defining genetic abnormalities and are categorized based on the differentiation of leukemia cells and how mature the cells are, which is similar to the French–American–British (FAB) classification and is mainly dependent on morphology and cytochemical criteria [3,4]. The forecast stratification and treatment decisions for patients with a normal karyotype are difficult due to the high degree of clinical heterogeneity; thus, identifying new predictive molecular markers is necessary to improve the classification and prognosis of AML.

The use of genetic molecular biomarkers has the potential to revolutionize the classification and treatment approaches for AML [5,6]. These biomarkers can provide insights into the molecular underpinnings of the disease, enabling the identification of subtypes with different biological behaviors and treatment responses. Consequently, the integration of molecular diagnostics into standard care is crucial for personalizing therapy and improving the outcomes of AML patients. Notably, the complexity of genomic data in AML is immense due to the extensive variability in genetic mutations and chromosomal alterations that characterize the disease. The genome of each patient contains a unique combination of genetic information, which creates a high-dimensional data space that is difficult to interpret with traditional statistical methods. The intricate patterns of gene expression and their interactions contribute to the heterogeneity of AML, making it more difficult to identify precise biomarkers and develop targeted therapies [7]. Given this complexity, machine learning has emerged as a pivotal tool in the analysis of genomic data [8,9]. Machine learning methods, such as feature selection algorithms, have revolutionized the field of bioinformatics by enabling the analysis of large-scale genomic and proteomic data [10,11,12,13]. These methods have been extensively applied to classify cancer subtypes based on genomic or proteomic data, providing valuable insights into tumor heterogeneity and personalized medicine. For instance, support vector machines (SVMs) or random forests (RFs) can be trained on gene/protein expression data from known cancer subtypes to learn patterns distinguishing them [14,15]. Feature selection techniques are often employed to identify a subset of gene/protein biomarkers that significantly contribute to classification accuracy, which reduces the dimensionality of the data and improves the accuracy of the classification [16]. The least absolute shrinkage and selection operator (LASSO) feature selection can handle the high-dimensional nature of genomic data and identify key biomarkers for AML classification due to its simplicity, efficiency, and strong feature selection capabilities, while Bayesian networks are particularly useful for modeling multiple interdependencies and updating predictions with new data based on the data after dimensionality reduction.

In this study, we investigated the expression of protein-coding genes in blood samples from AML patients and controls using The Cancer Genome Atlas (TCGA) and Genotype-Tissue Expression (GTEx) databases (detailed information is provided in the Methods section), and we subsequently applied the feature selection method of LASSO to select the optimal gene subset for classifying AML patients and controls as well as between a particular FAB subtype and other subtypes of AML. Among those involved, a number of genes were found to be correlated with the overall survival in AML patients by survival analysis. By identifying and choosing pertinent gene features, it is possible to effectively distinguish between different AML subtypes. This approach not only allows for improved diagnostic and treatment strategies but also has the potential to further our understanding of cancer heterogeneity and firmly support the morphological classification of AML defined by differentiation. Finally, the results of this study may aid in developing more precise categorization systems and personalized treatment plans for AML patients.

## 2. Materials and Methods

### 2.1. Data Acquisition and Preprocessing

The GTEx database is a comprehensive resource that provides information on gene expression patterns across various tissues in the human body [17]. This approach allows researchers to explore and analyze gene expression data to gain insights into tissue-specific gene regulation and its implications for human health and disease. TCGA aims to understand the molecular basis of cancer through the analysis of genomic and clinical data [18,19]. In this study, we derived gene expression data from the University of California-Santa Cruz (UCSC) Xena browser (https://xena.ucsc.edu/; accessed on 3 March 2024). Specifically, we extracted the gene expression RNAseq data of 337 blood samples (normal controls) and 151 samples of AML from the “TCGA TARGET GTEx” cohort (a combined cohort of TCGA, TARGET, and GTEx samples) in the UCSC Xena browser. The phenotypic information and survival data of the 151 AML samples were downloaded from the TCGA database (phenotype information: https://gdc-hub.s3.us-east-1.amazonaws.com/download/TCGA-LAML.GDC_phenotype.tsv.gz; accessed on 5 March 2024. survival data: https://gdc-hub.s3.us-east-1.amazonaws.com/download/TCGA-LAML.survival.tsv. accessed on 5 March 2024). The basic clinical information about the 151 AML patients is shown in Table 1.

The original dataset contains the expression patterns of 60,000+ genes in 487 samples (337 control samples and 151 AML cases). We screened 19,580 protein-coding genes from the original dataset by using the “EnsDb.Hsapiens.v75” package in R. Afterwards, the protein-coding genes with missing values were deleted, and a final dataset including the expression of 9932 genes in 487 samples was used for further analysis.

### 2.2. Using LASSO for Gene Feature Selection Between Different Groups

LASSO regression is an embedded algorithm and is a powerful method that helps perform the regularization and feature selection of given data [20,21]. It applies a penalty term to the regression coefficients, forcing some of them to be exactly zero, effectively selecting only the most important features. This helps to reduce overfitting and improve model interpretability. In LASSO regression, a 10-fold cross-validation model is used to increase the robustness of the model results. In this study, we employed MATLAB 2020 to perform LASSO regression to screen for the optimal candidate gene subset for the classification of different groups. The model of Lasso regression is as follows: y is the dependent variable, $x_{i}\in X$ is the independent variable, w is the regression coefficient, λ is the regularization parameter, and $\lambda \|w{\|}_{1}$ represents the L1 normal form of λ.

$$w=\underset{w}{*\mathrm{argmin}}\left(\sum_{i=1}^{N}{\left(y_{i}-w^{T}x_{i}\right)}^{2}+\lambda \|w{\|}_{1}\right)$$

### 2.3. Gene Functional Annotation

The functional annotation of candidate genes is a crucial step in understanding their biological roles and potential involvement in various cellular processes. In this study, we used the Database for Annotation, Visualization, and Integrated Discovery (DAVID) database to perform the functional annotation of the genes [22]. This comprehensive bioinformatics database was used to map our list of genes to corresponding functional categories, including Gene Ontology (GO) annotations for Biological Processes, Cellular Components, and Molecular Functions as well as Reactome Pathway mappings.

### 2.4. Classification Visualization

We performed t-distributed stochastic neighbor embedding (t-SNE), principal component analysis (PCA), and heatmap analysis to visualize the differences between the AML and control samples, as well as between one specific subtype and other subtype samples of AML. T-SNE is a nonlinear dimensionality reduction technique that can preserve local similarities between data points in high-dimensional space. Compared with traditional dimensionality reduction techniques, t-SNE employs an optimization process to find the best mapping that minimizes the Kullback–Leibler (KL) divergence between the conditional probabilities in the high-dimensional and low-dimensional spaces, offering a unique perspective on the structure and relationships within complex datasets. In the present study, the R package “Rtsne” was used to perform t-SNE to visualize the classification model. The R package “FactoMineR” and “pheatmap” were applied for PCA and heatmap analysis, respectively.

### 2.5. Construct Bayesian Network Using PC-Stable Algorithm

As a probabilistic graphical model for causal notation, the Bayesian network is a directed acyclic graph (DAG) that represents a set of variables and their conditional dependencies [23]. Bayesian networks are ideal for visualizing causal relationships and inferring probabilistic relationships between genes and classification nodes. The PC-stable algorithm is a modification of PC algorithms, and the modified version is much simpler while preserving existing soundness, completeness, and high-dimensional consistency. Herein, we employed the R (version 4.0.2)-package “pcalg” implemented by Kalisch to determine the causal correlations of the selected gene features.

### 2.6. Statistical Analysis

The statistical analysis was performed mainly using R 4.0.2 in this study. The differential expression gene analysis between the AML patients and controls was conducted using the Wilcoxon test (*p* < 0.05 indicated a significant difference). The Kaplan–Meier (K-M) curves for AML patients were analyzed using the “survival” and “survminer” packages in R. The cut-off points were used to divide the samples into “low” and “high” groups based on the gene expression level, with *p* < 0.05 indicating a significant difference between the two groups in K–M curves.

## 3. Results

### 3.1. Protein-Coding Gene Profiling Analysis of AML and Control Samples

**Figure 1:**
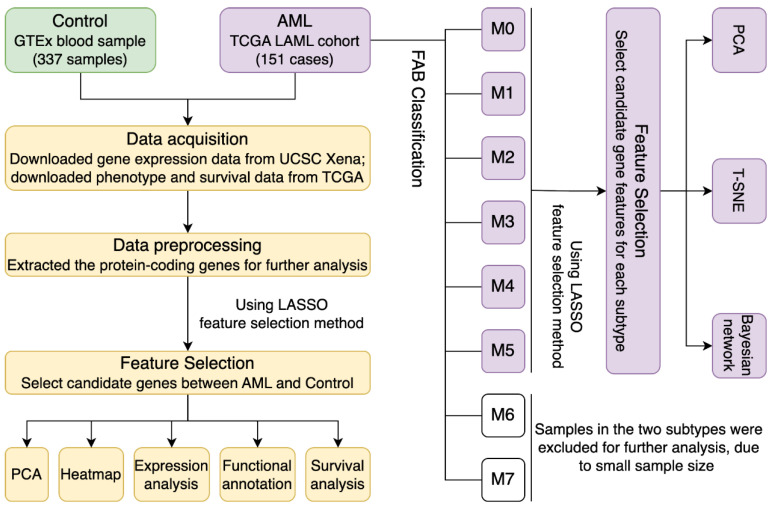
Workflow of the research.

**Figure 2:**
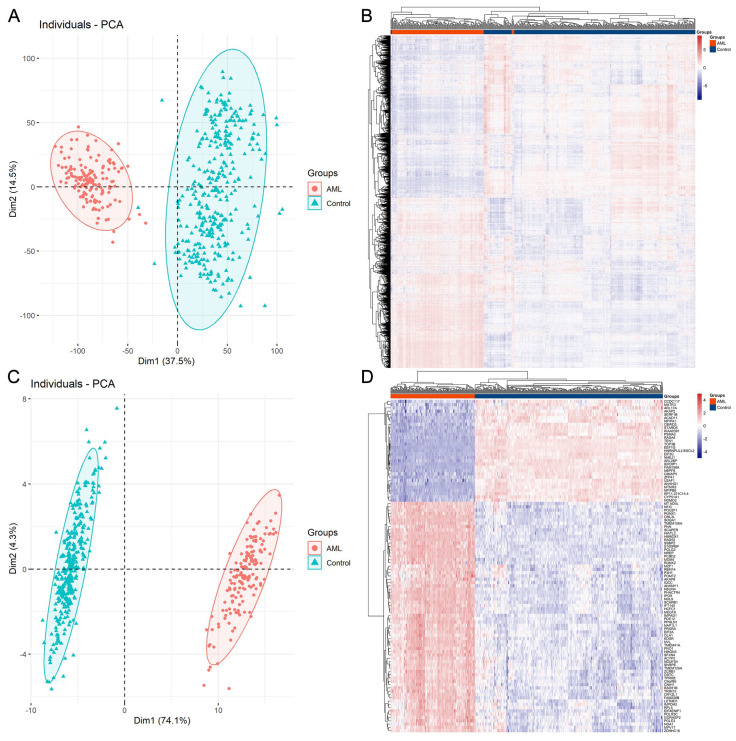
Visualization of PCA plots and heatmaps. (**A**,**B**) PCA plot and heatmap for AML and control samples based on the total of 9932 gene features. (**C**,**D**) PCA plot and heatmap for AML and control samples based on the 101 gene features selected by LASSO. The R packages “FactoMineR” and “pheatmap” were applied to PCA and heatmap analyses, respectively.

Figure 1 illustrates the workflow of the current study. The original datasets utilized in this study included more than 60,000 genes from 337 control samples and 151 AML samples. After data processing, a total of 9932 protein-coding genes remained for further research. According to the results from PCA (Figure 2A) and heatmap (Figure 2B) analysis, the gene expression patterns of the AML and control samples were relatively different based on the total 9932 gene features. Despite this, we applied the LASSO feature selection algorithm and selected the best gene subset, which helped to effectively distinguish AML patients from controls. The LASSO algorithm outputted a set of feature combinations containing 101 genes for discriminating between AML patients and controls. As displayed in Figure 2C,D, expression profiling of the 101 selected genes effectively distinguished between the two groups, with significantly improved performance compared to that of all 9932 genes. Thus, the application of feature selection methods to high-dimensional data aids in reducing dimensionality and improving model performance by selecting only the most relevant features, which provides insights into the significance of each gene in the diagnosis of AML.

### 3.2. Biological Analysis of the Selected Genes in AML

**Figure 3:**
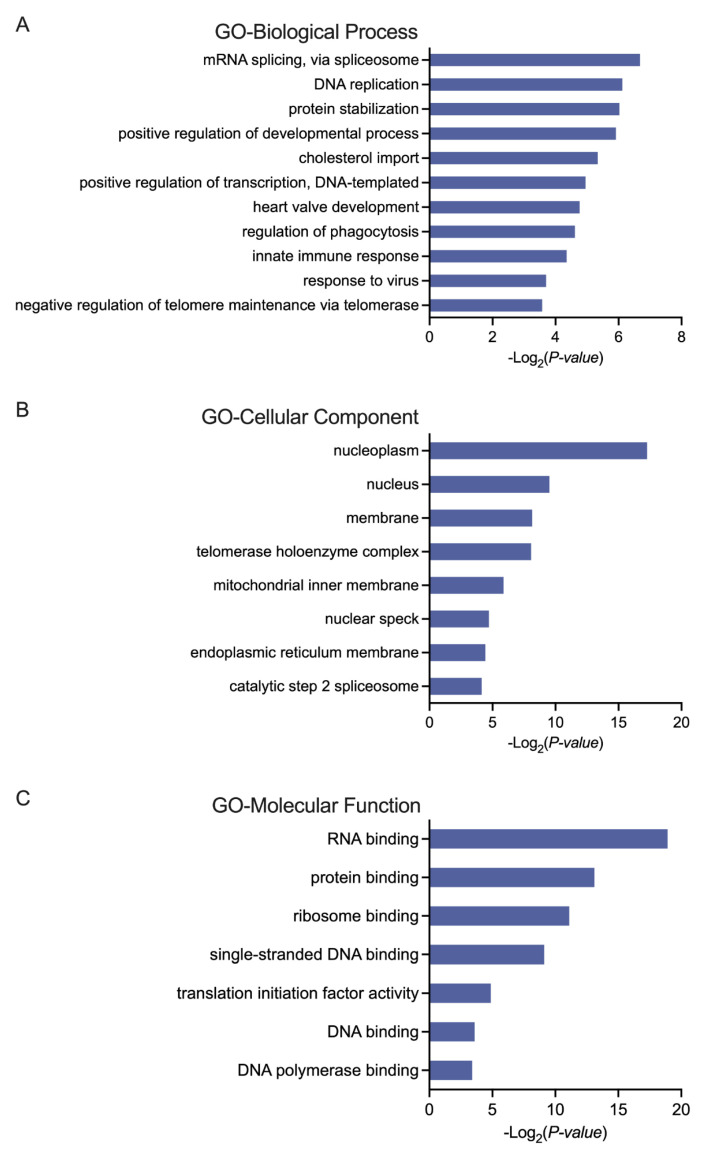
Gene Ontology (GO) annotations for the 101 gene features selected by LASSO. The GO terms include (**A**) Biological Process, (**B**) Cellular Component, and (**C**) Molecular Function. The DAVID database was used to assess the GO annotations.

**Figure 4:**
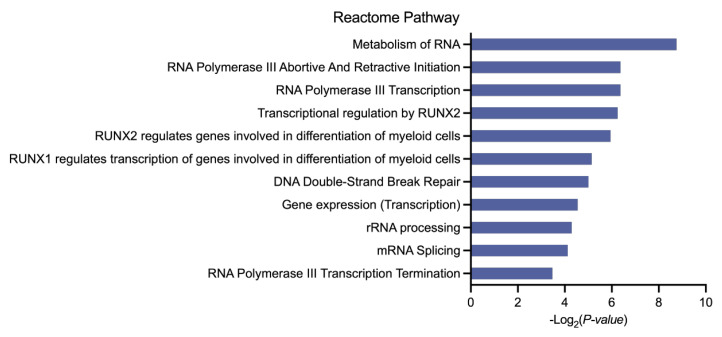
Reactome Pathway annotations for the 101 gene features selected by LASSO. The pathway annotations were analyzed using the DAVID database.

We previously selected 101 genes for better classification between AML and control samples and assumed that these gene features may provide insights into the underlying mechanisms for AML. According to the Wilcoxon test results, a distinct pattern of gene expression was observed in the two groups, with 70 genes up-regulated and 31 genes down-regulated in the AML patients compared to the control individuals (adjust. *p* < 0.0001, method = “bonferroni”) (Figure 2D). The detailed expression profiles of the 101 genes are displayed in Table S1. Using the DAVID gene annotation tool, we conducted a GO analysis of the 101 differentially expressed genes (DEGs). The DEGs were annotated for three subontologies, as indicated in Figure 3: Biological Process (BP), Cellular Component (CC), and Molecular Function (MF). For BPs, DEGs were primarily enriched in mRNA splicing, via spliceosome, DNA replication, and protein stabilization (Figure 3A). Regarding CCs, the DEGs were predominantly clustered in the nucleoplasm and nucleus (Figure 3B). In terms of the MF category, these genes were most enriched in RNA binding, protein binding, and ribosome binding (Figure 3C). Subsequently, REACTOME pathway analysis revealed that the identified DEGs were primarily implicated in the metabolism of RNA, RNA polymerase III abortive and retractive initiation, and RNA polymerase III transcription pathways. These findings aligned with the epigenetic alterations characteristic of AML [24,25,26] (Figure 4). Notably, specific genes like RUNX Family Transcription Factor 1 (*RUNX1*) and RUNX Family Transcription Factor 2 (*RUNX2*), have been demonstrated to play significant roles in both normal hematopoiesis and the development of blood cancers [27,28,29]. For instance, pathway annotation revealed that *RUNX1/RUNX2* regulates genes involved in the differentiation of myeloid cells. Our study results indicated that, in comparison to controls, the expression levels of *RUNX1* and *RUNX2* in AML patients had fold changes (FC) of 6.30 and 6.23, respectively (Table S1). These data suggest a potential involvement of these genes in the pathogenesis of AML.

### 3.3. Survival Analysis of Candidate Genes in AML

**Figure 5:**
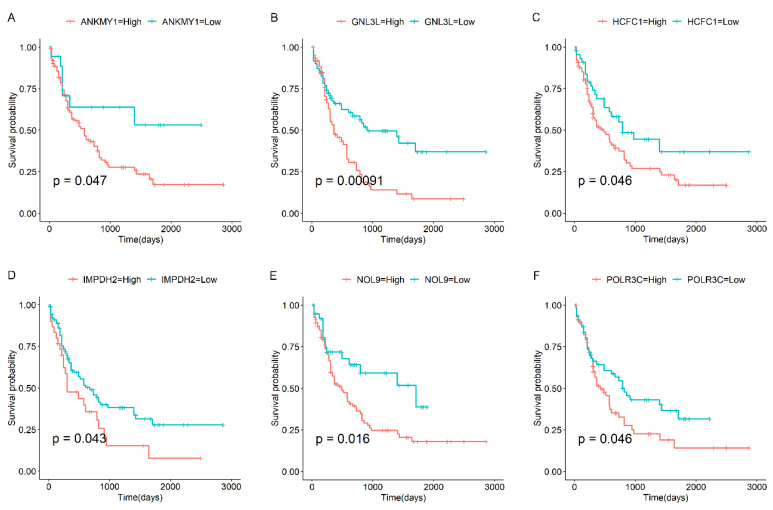
Prognostic analysis of candidate up-regulated genes. Shown here are the Kaplan–Meier curves for candidate up-regulated genes in AML compared to NC samples, including (**A**) *ANKMY1*, (**B**) *GNL3L*, (**C**) *HCFC1*, (**D**) *IMPDH2*, (**E**) *NOL9*, and (**F**) *POLR3C*. The Kaplan–Meier curves were analyzed using the “survival” and “survminer” packages in R.

**Figure 6:**
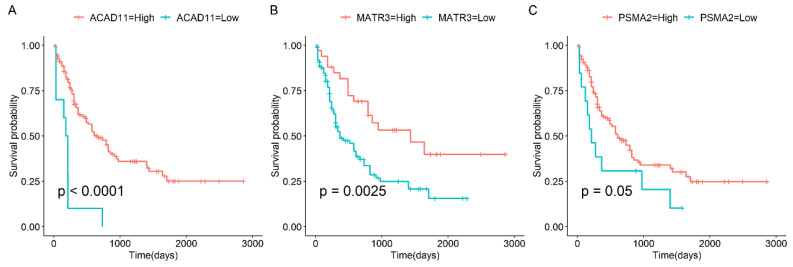
Prognostic analysis of candidate down-regulated genes. Shown here are the Kaplan–Meier curves for candidate down-regulated genes in AML compared to NC samples, including (**A**) *ACAD11*, (**B**) *MATR3*, and (**C**) *PSMA2*.

We then investigated the prognostic value of the selected genes, and we discovered that several candidate genes were significantly correlated with overall survival (OS) in AML patients. For instance, our findings indicated that the expression levels of G Protein Nucleolar 3 Like (*GNL3L*), Ankyrin Repeat and MYND Domain Containing 1 (*ANKMY1*), Host Cell Factor C1 (*HCFC1*), Nucleolar Protein 9 (*NOL9*), Inosine Monophosphate Dehydrogenase 2 (*IMPDH2*), and RNA Polymerase III Subunit C (*POLR3C*) were significantly up-regulated in AML patients compared to controls (Table S1). Elevated expression levels of the abovementioned candidate genes were linked to decreased OS in AML patients (Figure 5). Conversely, reduced expression of Acyl-CoA Dehydrogenase Family Member 11 (*ACAD11*), Matrin 3 (*MATR3*), and Proteasome 20S Subunit Alpha 2 (*PSMA2*) was associated with poorer OS outcomes (Figure 6). The majority of these genes are associated with the initiation or progression of AML. For example, the IMPDH2 inhibitor has been shown to be an effective treatment for MLL-fusion leukemia [30,31]. Overall, these genes have the potential to be predictive markers for the survival outcomes of AML patients and could be valuable targets [32,33]. Further research is warranted to validate these findings and explore the underlying mechanisms by which these genes influence patient survival.

### 3.4. Using the LASSO Method to Select Gene Subsets for Classifying AML Subtypes

**Figure 7:**
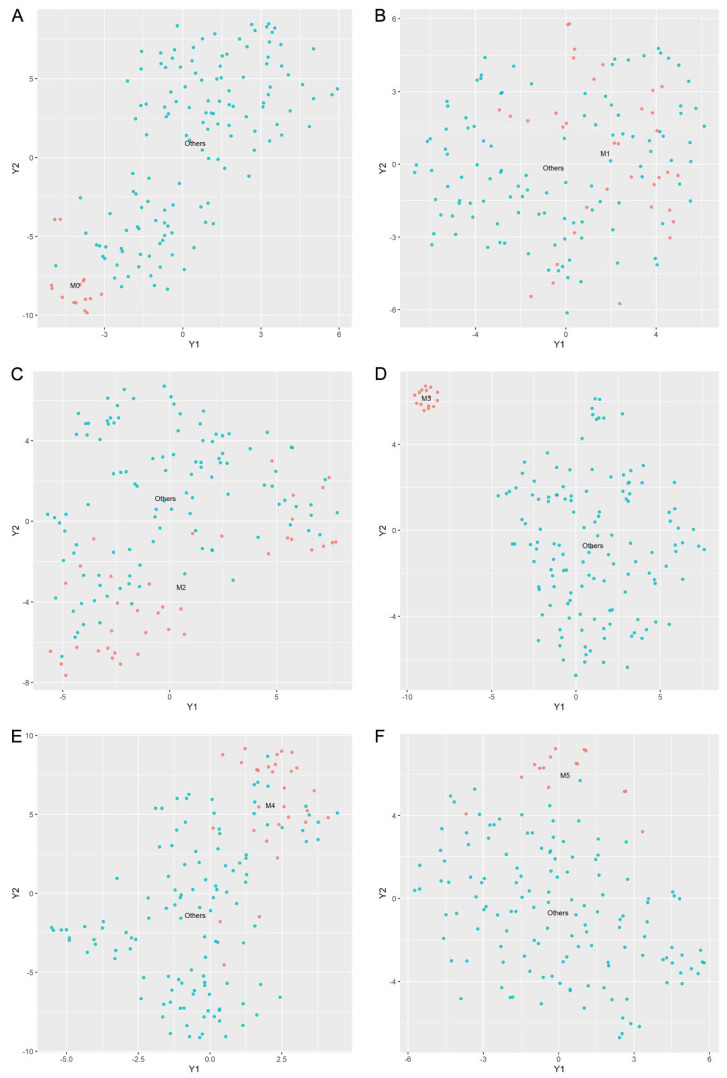
Visualization of t-SNE plots. The figure shows the t-SNE plots for (**A**) M0 and the other subtypes of AML, (**B**) M1 and the other subtypes of AML, (**C**) M2 and the other subtypes of AML, (**D**) M3 and the other subtypes of AML, (**E**) M4 and the other subtypes of AML, and (**F**) M5 and the other subtypes of AML.

Classifying AML subtypes is crucial for implementing personalized medicine and advancing our understanding of subtype-specific molecular mechanisms in AML. With the goal of identifying subsets of genes useful for accurately categorizing AML subtypes, we further utilized LASSO feature selection methods for gene selection. Due to the small sample size and low occurrence, we excluded the M6 and M7 subtypes from further research. As a result, we are concentrating our research on the remaining six subtypes, M0 through M5. Based on a total of 9932 gene expression profiles, t-SNE and PCA showed no distinct classifications among the remaining six cancer subtypes, except for M3 (Figure S1). We then employed the LASSO method for gene feature selection to distinguish between the different AML subtypes. Gene subsets containing 11, 7, 10, 35, 9, and 12 gene features were selected for classifying M0 versus others, M1 versus others, M2 versus others, M3 versus others, M4 versus others, and M5 versus others, respectively. As shown in Figure 7 and Figure S2, the subset of genes that were filtered out following feature selection helps to better discriminate between different AML subtypes, particularly for M0, M3, M4, and M5. Unfortunately, in cases of blasts with low differentiation levels and complicated cell types, such as M1 and M2, gene feature selection is unable to identify them effectively (Figure 7).

**Figure 8:**
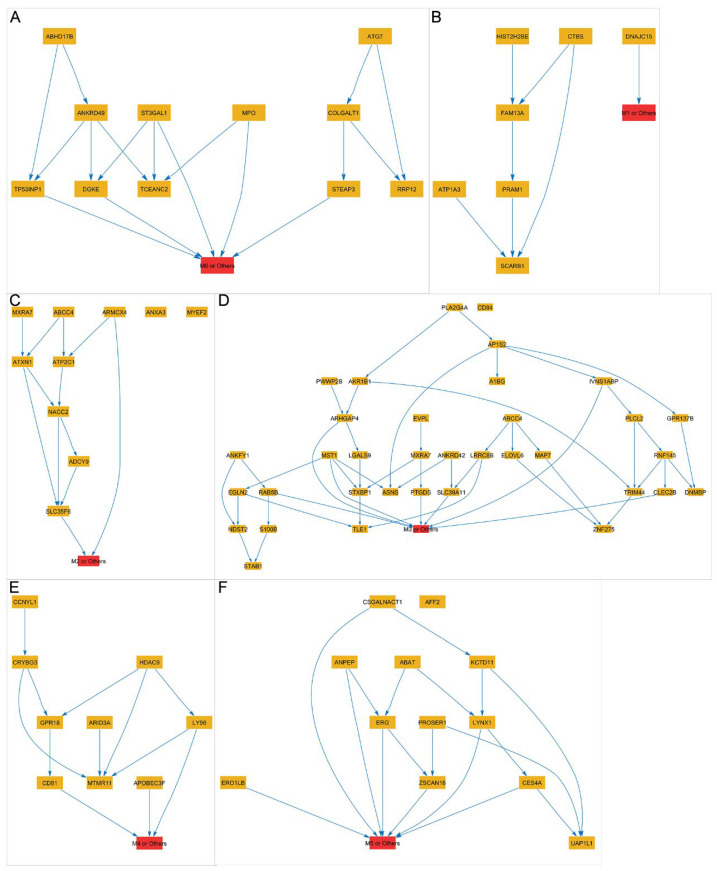
Bayesian networks (BN) of the selected gene features. Subsets of 11, 7, 10, 35, 9, and 12 genes were identified for classifying between (**A**) M0 and others, (**B**) M1 and others, (**C**) M2 and others, (**D**) M3 and others, (**E**) M4 and others, and (**F**) M5 and others, respectively. Shown here are the BN structures of the selected genes in each classification model.

To explore the interactions among the selected genes in each subset, a Bayesian network was constructed by defining the directed edges between gene nodes. As shown in Figure 8, the directionality of these edges represents causal relationships or effects between genes. By analyzing the direct effects of gene nodes within the Bayesian network, we can identify important genes that significantly impact the differentiation of one particular AML subtype from the others. The identification of these key genes is crucial for developing a better understanding of the pathogenesis of different subtypes of AML. Taken together, the abovementioned findings imply that the feature selection approach was successful in identifying key genes from the large-scale genomic dataset and that these selected genes may play critical roles in distinguishing between AML subtypes, potentially leading to more personalized and effective differentiation therapy strategies for AML patients. Further investigation of the functional importance of these genes could provide valuable insights into the underlying molecular mechanisms driving the development and progression of AML.

## 4. Discussion

Feature selection methods, as described in previous studies, play a pivotal role in identifying a subset of relevant genes that effectively discriminate between different cancer subtypes [34]. In this study, we utilized LASSO regression to screen out a subset of 101 genes for categorizing AML and control samples. Additionally, we identified gene subsets crucial for distinguishing a specific FAB subtype from other subtypes. This refined approach enhances our ability to distinguish between AML patients and healthy individuals, as well as between different AML subtypes, by recognizing and selecting relevant gene features. Furthermore, the selected gene subsets offer new prediction markers for AML patient prognosis, providing insights into the molecular-level pathogenic mechanisms underlying AML.

Compared to most other malignancies, AML exhibits a relatively lower somatic mutation burden according to genome sequencing [1,35]. Epigenetic dysregulation, which is central to the pathophysiology of AML, is a distinguishing feature of this disease [24,36]. Our findings also showed that the genes differentially expressed in AML are primarily associated with the metabolism of RNA and RNA polymerase Ⅲ (Pol Ⅲ) transcription. Pol Ⅲ is known to be highly specialized for the production of 5S rRNA, tRNA, and U6 snRNAs and is involved in the regulation of epigenetics [37,38]. These findings are consistent with the epigenetic changes observed in AML.

Genetic biomarkers play important roles in the early diagnosis, prognostic stratification, and surveillance of cancers, including AML. Current clinical guidelines for AML recognize three groups of cytogenetic risk—favorable, intermediate, and poor risk—through certain recurrent cytogenetic abnormalities and gene mutations, such as t(8; 21), t(15; 17), inv(16), inv(3), t(6; 9), 5q-, and *NPM1*, *FLT3*, *TP53,* and *CEBPA* mutations [3,39]. Nonetheless, according to the guidelines, almost 50% of adult patients with AML who have a normal karyotype are considered to be at intermediate risk [39,40]. The best therapeutic approaches for these patients have not been fully elucidated, and the treatment outcomes are heterogeneous. There is increasing evidence that the molecular analysis of genes, including those with mutations in *RUNX1*, *ASXL1*, and *MLL* as well as alterations in the expression levels of *BAALC* and *MN1*, can be used to identify a subgroup of poor-risk patients among those with normal cytogenic results [39,41,42,43]. Using LASSO regression, we identified a number of novel gene biomarkers related to the prognosis of AML in this study. For instance, we discovered that the conserved GTP-binding nucleolar protein GNL3L is linked to tumorigenesis and poor prognosis in patients with AML. The outcome was in line with recent research showing that GNL3L promoted AML cell proliferation and stimulated cytarabine resistance [32]. PSMA2 is a component of the 20S core proteasome complex, is a proteolytic degrader of most intracellular proteins, and is associated with prognosis. This protein can also form the PAN-PSMA2 fusion in Myelodysplastic Neoplasms (MDS) and progresses to AML [44]. Furthermore, a prior study demonstrated that PSMA2 may indirectly affect tumor cells through the immune microenvironment [45,46]. MATR3, a nuclear matrix protein that is thought to stabilize certain messenger RNA types [47], has also been shown to participate in creating the KMT2A-MATR3 fusion and accelerating the onset of acute leukemia [48]. Moreover, the IMPDH inhibitor FF-10501-01 has a potent therapeutic effect on aggressive AML with MLL rearrangements by excessively activating the TLR-VCAM1 pathway [30,31]. More research is necessary to validate these results and explore promising therapeutic targets for AML patients.

The FAB classification of AML poses a significant challenge, particularly in patients with negative cytochemical staining and in distinguishing between M1 and M2 or M2 and M4 [49]. Using the LASSO method for gene feature selection, we developed an improved strategy for distinguishing the M0, M3, M4, and M5 subtypes. The key genes we identified, which significantly impact the differentiation of a particular AML subtype from others, are likely associated with differentiation due to the varying degrees of differentiation arrest among the subtypes. These genes may serve as candidates for differentiation therapy. Therefore, additional research is needed to comprehensively understand the crucial roles played by these genes.

This study has several limitations. Certain AML subtypes, such as M1 and M2, posed challenges in effective gene feature selection, which may have impacted the robustness of the findings. Additionally, while the FAB classification provided a framework for subtype categorization in this study, it is acknowledged that the WHO classification offers a more contemporary and clinically relevant classification. Unfortunately, the WHO classification data were not available in the datasets utilized. This study was only focused on AML defined by differentiation, and future studies incorporating WHO classification will further enhance the clinical relevance of such analyses. Finally, further validation and functional studies are warranted to confirm the clinical relevance of the identified genes and their potential as therapeutic targets.

## 5. Conclusions

Our study underscores the application of machine learning, specifically the LASSO regression algorithm, for analyzing the complex genomic data of AML patients. We successfully identified a subset of 101 genes that effectively distinguished between AML and control samples. Importantly, the identified genes not only are valuable for diagnosis but also demonstrate prognostic significance, with certain genes correlating with the overall survival outcomes of AML patients. Moreover, the genomic signature was further analyzed using LASSO to classify different AML subtypes, which may help to better understand the molecular heterogeneity within AML. Overall, our study provides insights for more precise categorization and personalized treatment strategies for AML.

## Figures and Tables

**Table 1:** Clinical characteristics of the AML cohort.

Clinical Characteristics	AML Cohort
Age	
N/Range	151/(21–88)
Average	54.17 ± 16.07
Gender (N)	
Male	83
Female	68
Race (N)	
Asian	1
Black	13
White	135
Not reported	2
Subtype (N)	
M0	15
M1	35
M2	38
M3	15
M4	29
M5	15
M6	2
M7	1
Not reported	1
OS_status (N)	
Alive	52
Dead	80
Not reported	19
OS_time (Days)	
Alive	927.73 ± 730.63
Dead	414.50 ± 385.15

N: number of cases; OS: overall survival.

## Data Availability

The gene expression RNAseq data were downloaded from the UCSC Xena browser (https://xena.ucsc.edu/). The phenotypic information and survival data of the AML samples were downloaded from the TCGA database (phenotype information: https://gdc-hub.s3.us-east-1.amazonaws.com/download/TCGA-LAML.GDC_phenotype.tsv.gz; survival data: https://gdc-hub.s3.us-east-1.amazonaws.com/download/TCGA-LAML.survival.tsv. Both datasets were retrieved on 5 March 2024).

## Supplementary Materials

The following supporting information can be downloaded at: https://www.mdpi.com/article/10.3390/biomedicines13051067/s1, Figure S1: Visualization of t-SNE and PCA plots. (A) T-SNE plot of the six subtypes of AML based on the total 9932 gene features. (B) PCA plot of the six subtypes of AML based on the total 9932 gene features; Figure S2: Visualization of PCA plots. The figure shows the PCA plots for (A) M0 and other subtypes of AML, (B) M1 and other subtypes of AML, (C) M2 and other subtypes of AML, (D) M3 and other subtypes of AML, (E) M4 and other subtypes of AML, and (F) M5 and other subtypes of AML; Table S1: Excel file containing the detailed expression profiling of the selected 101 genes in AML patients and controls, related to Figure 2.

## Abbreviations

AML	Acute myeloid leukemia
LASSO	Least Absolute Shrinkage and Selection Operator
HSCs	Hematopoietic stem cells
WHO	World Health Organization
FAB	French–American–British
SVM	Support vector machines
RF	Random forests
GTEx	Genotype-Tissue Expression
TCGA	The Cancer Genome Atlas
DAVID	Database for Annotation, Visualization, and Integrated Discovery
GO	Gene Ontology
t-SNE	t-distributed stochastic neighbor embedding
PCA	Principal component analysis
DEGs	Differentially expressed genes
BP	Biological processes
CC	Cellular component
MF	Molecular function
Pol III	RNA polymerase III
