# Supplementary material for: Acute Myeloid Leukemia Genome Characterization Study and Subtype Classification Employing Feature Selection and Bayesian Networks
Source: Biomedicines. 2025 Apr 28;13(5):1067. doi: 10.3390/biomedicines13051067 (PMC12109106; doi:10.3390/biomedicines13051067)
Supplement: Supplementary file 1 [file biomedicines-13-01067-s001.zip › biomedicines-3563400-supplementary/Supplementary Figures.docx]

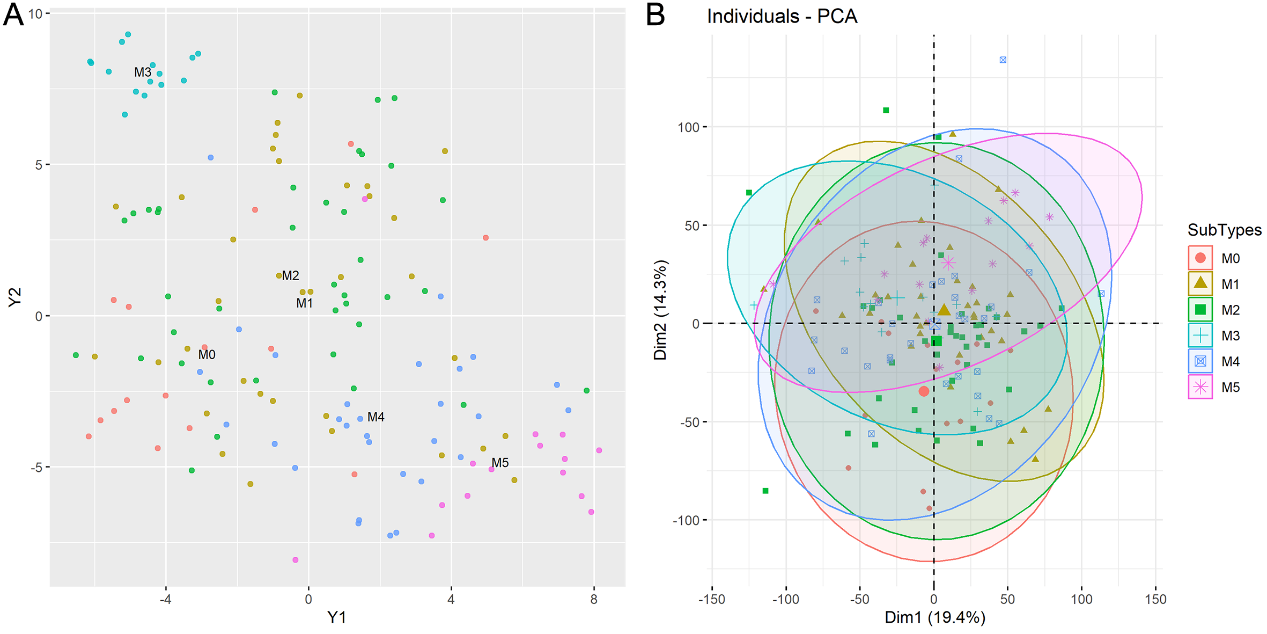


**Figure S1**. Visualization of t-SNE and PCA plots. (A) T-SNE plot of the six subtypes of AML based on the total 9,932 gene features. (B) PCA plot of the six subtypes of AML based on the total 9,932 gene features.


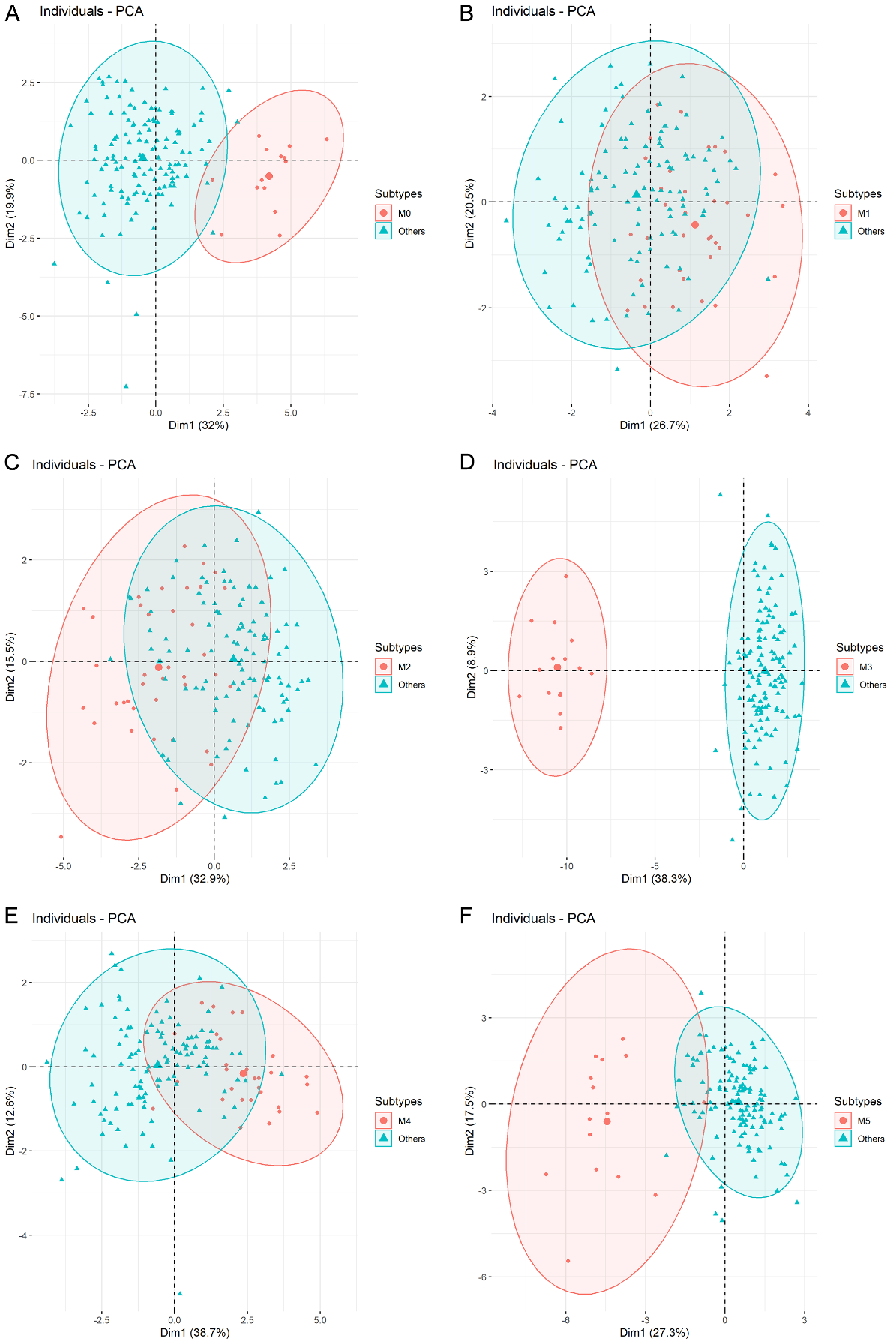


**Figure S2**. Visualization of PCA plots. Here shows the PCA plots for (A) M0 and other subtypes of AML, (B) M1 and other subtypes of AML, (C) M2 and other subtypes of AML, (D) M3 and other subtypes of AML, (E) M4 and other subtypes of AML, and (F) M5 and other subtypes of AML.
